# Trait-Mediated Variation in Plant Interactive Roles Within Plant–Floral Visitor Networks

**DOI:** 10.3390/plants15020289

**Published:** 2026-01-17

**Authors:** Fernanda Baena-Díaz, Brenda Ratoni, Carlos Pinilla Cruz, Ricardo Ayala, Wesley Dáttilo

**Affiliations:** 1 Red de Ecoetología, Instituto de Ecología AC (INECOL), Veracruz 91073, Xalapa, Mexico; brendam_rg@outlook.com; 2 Department of Zoology, Faculty of Sciences, Charles University, 12800 Prague, Czech Republic; crlspcruz@gmail.com; 3 Estación de Biología Chamela, Instituto de Biología, Universidad Nacional Autónoma de México (UNAM), San Patricio 48895, Jalisco, Mexico; rayala@ib.unam.mx

**Keywords:** ecological networks, functional characteristics of plants, plant–pollinator interactions, tropical coastal, trait-mediated interactions ecosystems

## Abstract

Plant–pollinator interactions are essential to ecosystem functioning, yet the mechanisms that determine why some plant species become highly connected within interaction networks remain insufficiently understood, particularly in tropical coastal systems. Here, we examine how multiple plant traits predict the interactive role of species within a bee–plant network in a coastal ecosystem in the Gulf of Mexico. Using an existing dataset comprising 35 plant species and 47 bee species, we quantified each plant’s interactive role through species degree, betweenness, and closeness centrality. We then evaluated how six traits (i.e., flower number, flower size, flower color, number of stamens, plant height, and life form) influence these network positions. Our results show that four traits significantly predicted plant interactive roles. Plants surrounded by more open flowers and those with larger flowers interacted with a greater diversity of bee species, indicating that resource detectability and accessibility strongly shape visitation patterns. Herbaceous species also exhibited higher interactive roles than woody plants, likely due to their rapid growth, abundant and synchronous flowering, and predictable resource availability in dynamic coastal environments. Additionally, yellow-flowered species received disproportionately more visits and achieved higher interactive roles, consistent with known sensory biases of bees toward yellow wavelengths. In contrast, plant height and stamen number showed no detectable influence on network position. Overall, our findings demonstrate that a combination of vegetative and floral traits (particularly those signaling abundant, accessible, and visually detectable resources) drives the emergence of key plant species within bee–plant networks. Integrating plant traits with network metrics provides a powerful framework for identifying species that sustain pollinator diversity and for predicting community responses to environmental change.

## 1. Introduction

Mutualistic interactions are fundamental to ecosystem functioning, as they regulate key ecological processes and mediate the persistence of biodiversity across terrestrial ecosystems [1,2]. Among these interactions, plant–pollinator relationships represent one of the most important ecosystem services, because they directly sustain plant reproduction and, indirectly, the structure and diversity of entire biological communities. It is estimated that more than 80% of flowering plant species rely, at least partially, on animal pollination to achieve successful reproduction, highlighting the pervasive influence of pollinators on ecosystem dynamics and resilience [3,4,5]. In recent decades, researchers have increasingly used tools derived from network theory to study plant–pollinator systems at the community level. These studies show that plant–pollinator interaction networks are structured by non-random interaction patterns, driven by species abundance, evolutionary histories, and abiotic conditions such as climate and resource availability [6,7,8]. Despite these general patterns, networks remain highly context-dependent at local and seasonal scales [8,9,10,11].

Local variation in plant communities and flowering dynamics shapes pollinator behavior and interaction networks. For example, floral abundance and phenology directly influence a plant’s network role and pollinator diversity by altering its attractiveness and temporal availability [12,13]. These dynamics link overall system stability to the interplay of plant traits, community context, and pollinator responses, highlighting the need for trait-based approaches. Indeed, while networks show community patterns, the underlying mechanisms are driven by species-level traits that guide pollinator attraction and consistent foraging choices [14,15,16,17,18,19]. Although key attractive traits such as flower number, size, nectar, odor, color, and pollen presentation are well documented [15,20,21], and plant strategies to enhance fitness through these traits are widely described [19,22], most studies remain narrow in scope. Due to the unique nature of each interaction, research on how traits affect attraction often focuses on only one or a few plant and pollinator species [15,23,24].

Studies at the community level have associated different plant traits with plant–pollinator interactions [25,26,27,28,29,30]. For example, in the work by Campbell and collaborators [30], experimental plots testing different corolla sizes showed associations with different insect groups. The study by Carvalheiro et al. [26] found that plants with higher resources, like flower number and content of nectar sugar, had a higher probability of influencing other plants by sharing pollinators. Other studies have established that plant-pollinator interactions are modulated by factors like species richness and visit frequency [31,32], but they mostly exclude species-level network metrics such as centrality (the relative importance or influence of a species within the network) and degree of interactions (the number of different interaction partners a species has) (but see [33]). Further studies have focused on how plant similarity explain pollinator overlap and thus network properties such as nestedness (the tendency for species with fewer interaction partners to interact with a subset of the partners of species with more interactions) and modularity (the degree to which the network is organized into groups of species that interact more strongly within groups than between them) [34,35,36,37]. Despite recognizing that both traits and network structure influence pollination outcomes, we still lack integration between these approaches, particularly regarding how measurable plant traits can predict a species’ interactive role within pollination networks [38]. The interactive role can be defined as the extent of a species’ direct and indirect connections within a network [39,40], reflecting not only how it interacts with pollinators but also how it functionally links different components of the community. Species with a central interactive role can facilitate pollinator sharing among plants, contribute disproportionately to network cohesion, and buffer the system against the loss of other species, thereby enhancing overall stability and resilience [39,40]. Understanding the determinants of a species’ interactive role is therefore crucial for predicting the functional consequences of changes in plant communities and for conserving the integrity and persistence of pollination networks. Furthermore, geographical and taxonomical biases persist in the study of plant–pollinator interaction networks [41]. Most research has been concentrated in temperate systems, leaving tropical ecosystems, such as coastal areas, underrepresented. These tropical coastal systems face unique environmental pressures, including high species diversity, strong seasonal variation, and specific abiotic conditions, that may shape trait–network relationships in ways not observed in temperate environments [42]. Investigating how plant traits determine species’ interactive roles in these contexts is therefore essential to broaden our understanding of network functioning, species’ contributions to ecosystem stability, and the maintenance of biodiversity in highly dynamic ecosystems.

In this study, we aim to explore whether variation in plant traits related to plant reproduction and bee pollinator attraction mediates the interactive role of plants in a coastal tropical ecosystem. We selected four floral traits based on evidence of their relevance to pollinator attraction (i.e., flower color and flower size) and reward economics (i.e., flower number and number of stamens), as well as two vegetative traits related to resource allocation constraints (i.e., life form and plant height). Considering these traits, we expect the interactive role of plants to be associated with different trait combinations, as pollinators are likely to exhibit diverse preferences. Specifically, we anticipate that flower number and flower size will be positively associated with pollinator visitation [12,25]. However, generating predictions for the remaining traits remains challenging due to the limited empirical information available on the specific trait preferences of pollinators within this community.

## 2. Materials and Methods

### 2.1. Study Area

The study was conducted at the Centro de Investigaciones Costeras La Mancha (CICOLMA), a protected natural reserve located in the state of Veracruz, Mexico, along the Gulf of Mexico coast (19°35′12″–19°36′18″ N and 96°22′18″–96°23′24″ W). CICOLMA encompasses a mosaic of coastal and lowland ecosystems that reflect strong environmental heterogeneity over relatively short spatial scales. The reserve includes several distinct vegetation types, such as tropical deciduous forest, coastal dune systems, scrubland, floodplain deciduous forest, mangrove forest, and pioneer dune vegetation, which together support high levels of plant and animal diversity and a wide range of ecological interactions [43,44,45]. The regional climate is classified as warm sub-humid, with mean annual temperatures ranging from 22 to 26 °C and a marked seasonality in rainfall. Annual precipitation averages approximately 1500 mm, with most rainfall concentrated during the summer months, contributing to pronounced temporal variation in resource availability and ecological processes across the different habitats within the reserve [43,45].

### 2.2. Database and Plant Traits

To estimate the interactive role of each plant species within the plant–bee network and to evaluate how these roles are associated with plant functional traits, we used a database previously compiled by our research group [46]. This dataset was obtained from intensive field sampling conducted over 40 days, totaling approximately 200 h of systematic flower-visitor observations. During the sampling period, focal plant individuals were monitored to record bee visitation, and all visits by bee species were quantified, comprising a total of 998 interactions. For each focal plant, the mean number of open flowers was also recorded within a 5 m radius, providing a measure of local floral availability that accounts for variation in resource abundance. The database comprised flowering plant species, collected and identified in the herbarium XAL at the Institute of Ecology A.C. (INECOL), and included detailed information on the frequency of visits by different bee species, collected and identified in the lab by a specialist taxonomist of our team (RA). In addition, a suite of functional plant traits potentially relevant to pollinator attraction and accessibility was measured. These traits included five floral traits: corolla width, corolla tube length, pistil length, and the lengths of the shortest and longest stamens, which together capture variation in floral morphology and reproductive organ architecture. A vegetative trait, plant height, was also included as a proxy for plant visibility and spatial positioning within the community.

To further characterize species’ functional attributes, we complemented the original dataset with additional trait information obtained from multiple published sources [47,48,49]. These complementary traits included plant life form (classified as herbaceous, tree, or shrub), flower color as perceived by humans, and the number of anthers, which may influence pollen availability and reward levels for floral visitors. Together, this comprehensive set of interaction data and plant traits allowed us to assess how variation in plant functional characteristics relates to their interactive roles within the plant–bee network.

### 2.3. Estimation of Interactive Role

The interactive role of each plant species within the plant–pollinator network was quantified using the complete weight plant–pollinator interaction matrix and a set of complementary network descriptors calculated at the species level. Specifically, we estimated three widely used metrics that capture different aspects of species importance in bipartite networks: species degree, betweenness centrality, and closeness centrality. All metrics were computed using the bipartite package in R [50].

Species degree (k) represents the total number of bee species interacting with a given plant species and provides a direct measure of interaction richness or partner diversity [51]. Betweenness centrality quantifies the extent to which a plant species lies on the shortest paths connecting pairs of species in the network, thereby reflecting its potential role as a connector that facilitates indirect interactions and the flow of ecological effects across the network [51]. Closeness centrality measures the average shortest path length between a focal plant species and all other species in the network, indicating how centrally located a species is and how rapidly it can influence, or be influenced by, other species within the network structure [52]. Because these three descriptors capture related but not independent dimensions of network structure and were found to be highly correlated, we integrated them into a single synthetic measure of species importance using principal component analysis (PCA). The first principal component (PC1) explained 81% of the total variance among metrics and was therefore retained as an overall index of a plant species’ interactive role within the network. Higher PC1 scores correspond to plant species that interact with a larger number of bee species and occupy more central positions in the network, both through direct interactions and through indirect connections mediated by shared partners [39,53]. This composite index allowed us to summarize multiple aspects of network centrality into a single, biologically meaningful variable for subsequent analyses.

### 2.4. Trait Dimensionality Reduction

Because several floral traits were also strongly correlated, we reduced trait dimensionality using principal component analysis (PCA) to avoid multicollinearity and to synthesize variation in floral morphology into a smaller number of independent axes. The PCA included four floral traits directly related to floral size and accessibility: corolla width, corolla tube length, and the lengths of the shortest and longest stamens. Prior to analysis, all variables were standardized to unit variance to ensure that traits measured on different scales contributed equally to the ordination.

Pistil length was excluded from the analysis due to missing values for a subset of species, which would otherwise have substantially reduced the sample size. After this exclusion, the final dataset comprised 35 plant species. The first principal component (PC1) explained 92% of the total variance among the included floral traits and was therefore retained for subsequent analyses. This axis was interpreted as a “flower size” gradient, as all trait loadings were positive and of similar magnitude, indicating that PC1 captured coordinated increases in overall floral dimensions rather than variation in floral shape or proportionality [54]. By summarizing correlated floral traits into a single composite variable, this approach allowed us to retain biologically meaningful information on floral morphology while simplifying subsequent statistical analyses and improving interpretability.

### 2.5. Data Analysis

To evaluate whether plant functional traits predict the interactive role of each species within the plant–bee network, we fitted a linear model using the plant’s interactive role, expressed as PC1 scores derived from network metrics, as the response variable. As explanatory variables, we included a set of floral and vegetative traits expected to influence pollinator attraction, accessibility, and interaction frequency. Specifically, the predictors were the mean number of open flowers, flower size (PC1 scores from the floral trait PCA), flower color, number of stamens, plant height, and life form.

Categorical predictors were grouped to ensure adequate sample sizes within each level and to facilitate biological interpretation. Flower color was classified into four categories: white (including whitish flowers), yellow, purple (including blue and pink hues), and others, which encompassed less common colors such as greenish and brownish tones. Plant life form was classified into three categories: herbaceous species, woody species (shrubs and trees), and others, including rushes and vines. The number of stamens was grouped into three classes (<10, 10, and >10), reflecting broad differences in pollen production potential.

To meet model assumptions, continuous predictors were transformed prior to analysis. The flower size axis was square-root transformed, and the mean number of open flowers was log-transformed to improve normality and reduce heteroscedasticity. Model residuals were visually inspected to confirm compliance with assumptions of linearity and homoscedasticity. When significant effects of categorical predictors were detected, post hoc pairwise comparisons among factor levels were conducted using estimated marginal means implemented in the emmeans package [55]. All statistical analyses were performed in R version 4.5 [56].

## 3. Results

**Figure 1:**
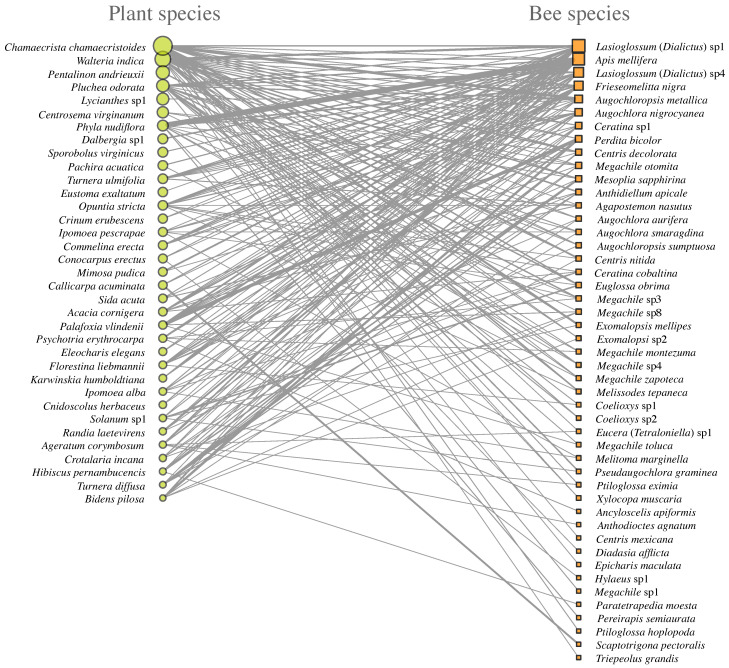
Plant–floral visitor network sampled at the Centro de Investigaciones Costeras La Mancha (CICOLMA), Mexico. Nodes represent plant and bee species, and links indicate recorded visitation interactions. Plant and bee nodes are distinguished by color, and link width reflects interaction frequency.

**Figure 2:**
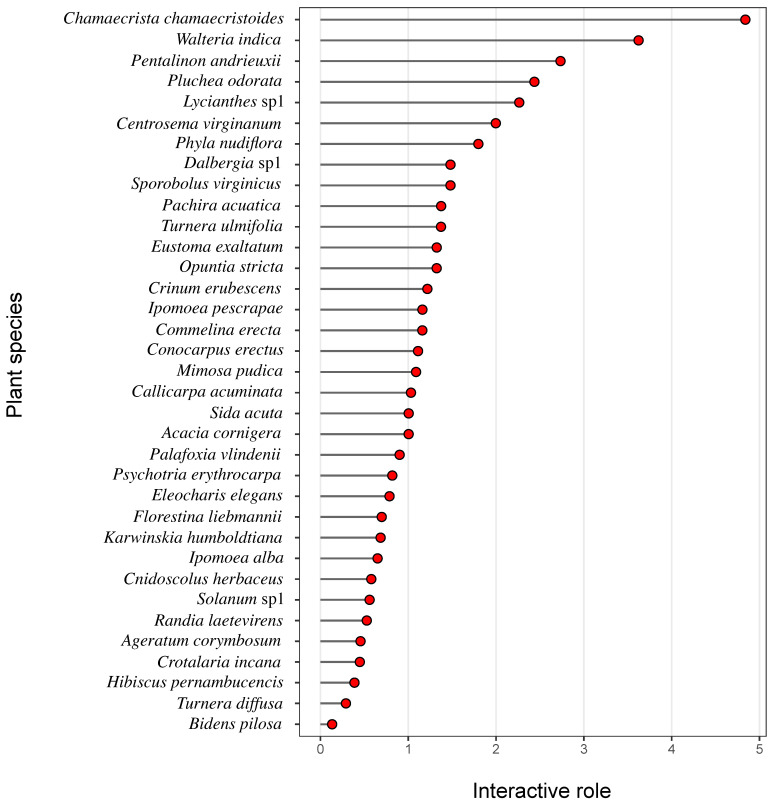
Interactive role of plant species in the plant-floral visitor network sampled in the Centro de Investigaciones Costeras La Mancha (CICOLMA), Mexico. Plant species are ranked from the highest to the lowest interactive role value.

The 35 plant species analyzed belonged to 21 plant families, with Asteraceae and Fabaceae being the most species-rich families, each represented by five species. In terms of growth form, herbaceous plants comprised most of the community (52.7%), followed by woody species (38.8%), while shrubs and vines together accounted for 9.3% of the sampled species (Figure 1). Considerable variation was observed in the interactive role of plant species within the plant–bee network. Among all species, *Chamaecrista chamaecristoides* (Fabaceae) exhibited the highest interactive role, followed by *Waltheria indica* (Malvaceae). Both species interacted with more than 20 bee species, highlighting their central positions within the network (Figure 2). The flower visitor assemblage comprised 47 bee species distributed across five families. Apidae was the most diverse family, represented by 17 genera, followed by Halictidae with six genera, Megachilidae with four genera, Colletidae with two genera, and Andrenidae with a single genus (Figure 1). The most frequently recorded visitor was a species of *Lasioglossum (Dialictus)* sp1 (Halictidae), followed by *Apis mellifera* (Apidae), both of which interacted with a large number of plant species.

**Figure 3:**
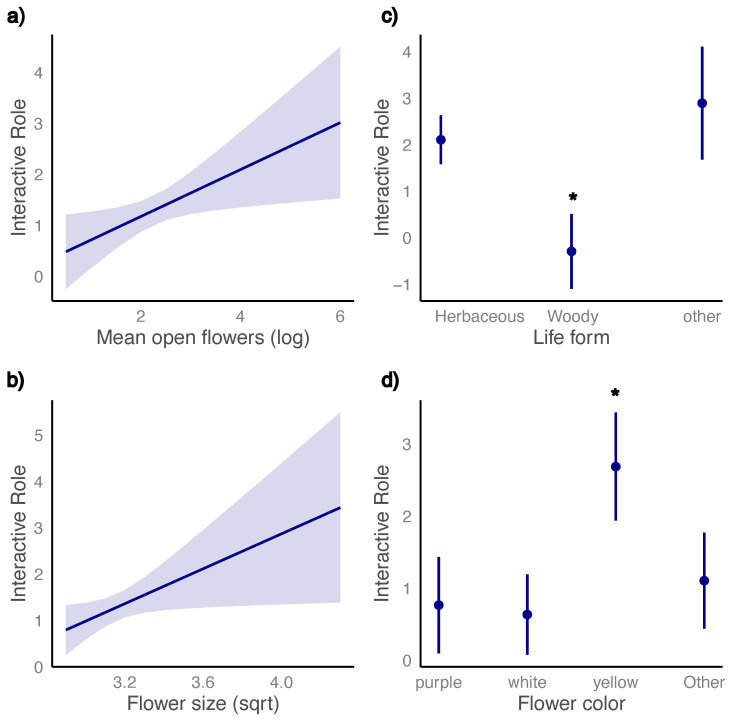
Model estimates (±95% confidence intervals) showing the relationship between plant traits and the interactive role of plant species: (**a**) means number of open flowers, (**b**) flower size (PC1), (**c**) life form (Woody = trees and shrubs; Other = rushes and vines), and (**d**) flower color. Asterisks indicate significant post hoc contrasts (*p* < 0.001).

Linear model results revealed that four plant traits were significantly associated with the interactive role of plant species. Specifically, species with a greater mean number of open flowers exhibited higher interactive role values (χ^2^ = 6.10, d.f. = 1, *p* = 0.013; Figure 3a). Similarly, plants characterized by larger flowers, as represented by the flower size PCA axis (explaining 92% of the variance), showed significantly higher interactive roles (χ^2^ = 4.88, d.f. = 1, *p* = 0.027; Figure 3b).

Interactive role also differed significantly among life forms, with herbaceous species and those classified as rushes or vines exhibiting higher interactive roles than woody species (shrubs and trees) (χ^2^ = 18.9, d.f. = 2, *p* < 0.001; Figure 3c). In addition, flower color was significantly related to species’ interactive role, with yellow-flowered species presenting higher values than species with white, purple, or other flower colors (χ^2^ = 18.6, d.f. = 3, *p* = 0.0003; Figure 3d). In contrast, plant height showed no significant relationship with interactive role (χ^2^ = 0.048, d.f. = 1, *p* = 0.82), nor did the number of stamens (χ^2^ = 0.71, d.f. = 2, *p* = 0.69), indicating that these traits did not contribute to explaining variation in species’ positions within the network.

## 4. Discussion

Bee–plant interactions sustain the production of seeds and fruits in both natural and agricultural systems, thereby maintaining ecosystem functioning and contributing directly to food security worldwide [57]. The strength and stability of these interactions depend not only on the presence of pollinators but also on the frequency, diversity, and identity of bee visitors. As a result, plants are expected to exhibit floral and vegetative traits that enhance detectability, accessibility, and attractiveness to pollinators, ultimately increasing their reproductive success [58,59]. Despite the recognized importance of trait-mediated interactions, most previous studies have examined the effects of individual floral traits in isolation or have been conducted predominantly in temperate systems. This has limited our understanding of how multiple floral and vegetative traits jointly shape the importance of plant species within interaction networks, particularly in tropical coastal ecosystems characterized by high environmental heterogeneity and strong seasonal dynamics. Moreover, relatively few studies have explicitly linked plant functional traits to network-based measures of species importance, leaving uncertainty about which traits most strongly predict plant interactive roles when evaluated simultaneously within real community contexts. Our results help to address this gap by showing that a combination of floral display and growth-form traits is closely associated with plant interactive roles in a coastal plant–bee network along the Gulf of Mexico. Specifically, plant species surrounded by a greater number of open flowers, with larger flowers and yellow coloration, and exhibiting herbaceous life forms occupied more central positions in the interaction network and were visited by a higher diversity of bee species. These traits likely increase visual detectability and foraging efficiency for bees, thereby promoting repeated and diversified visitation. In contrast, vegetative size and the number of stamens did not influence plant roles within the network, suggesting that floral visibility and accessibility may be more important than plant stature or potential pollen quantity in structuring interactions at the community scale.

Taken together, these findings indicate that specific combinations of floral and vegetative traits determine which plant species emerge as key nodes within bee–plant networks. By acting as central and highly connected resources, such plant species may play a disproportionate role in sustaining local bee diversity and maintaining the structure and resilience of pollination networks in tropical coastal ecosystems.

Pollinators often rely heavily on visual cues to locate and assess floral resources, as such cues can reliably signal both the quantity and accessibility of rewards offered by flowers [27,59,60]. Floral abundance and display size are therefore expected to play a central role in shaping pollinator foraging decisions. In agreement with previous studies showing that areas with greater floral cover receive higher visitation rates [61], we found that plant species surrounded by a larger number of open flowers not only attracted more individual bee visits but also interacted with a greater diversity of bee species. This translated into a markedly higher interactive role for these species within the plant–bee network, highlighting the importance of floral display at the community scale.

In addition to floral abundance, flower size emerged as a strong predictor of plant importance within the network. Plant species with larger flowers received disproportionately more visits, consistent with empirical evidence indicating that larger floral structures often provide greater quantities of pollen and nectar, which constitute the primary nutritional rewards for bees [28,29]. Beyond reward quantity, larger flowers may also enhance physical accessibility by accommodating bees of a wide range of body sizes, from small halictidae species to larger apidae bees. This structural inclusiveness allows both small and large visitors to land, access floral rewards, and forage efficiently [29], thereby increasing the diversity of pollinators able to interact with a given plant species. By simultaneously increasing detectability, reward availability, and accessibility, plants with many and larger flowers are more likely to be repeatedly visited by multiple bee species. This, in turn, elevates their connectivity and centrality within the interaction network. Such highly connected plant species act as hubs that facilitate pollen transfer across the community, enhancing reproductive success at the plant level while also supporting a diverse assemblage of pollinators. Thus, our results suggest that the combined effects of high floral display and large flower size favor a reproductive strategy in which plants emerge as key species with high interactive roles in bee–plant networks [62,63]. Further studies should integrate visitation data with pollination effectiveness metrics to better resolve how trait-mediated patterns of visitation translate into plant reproductive success, strengthening the link between network structure, species’ interactive roles, and ecosystem functioning [64].

Herbaceous plants acted as central species within the flower–visitor network studied, likely due to their short life cycles, rapid growth rates, and often synchronous and abundant flowering, which together generate predictable and readily accessible floral resources throughout the flowering season [65]. These traits make herbaceous species particularly reliable foraging options for a wide range of bee taxa, especially in dynamic coastal environments such as those along the Gulf of Mexico, where resource availability can fluctuate markedly in response to seasonal variation, flooding regimes, and salinity gradients. In this context, herbaceous plants function as “resource hubs,” concentrating floral rewards in space and time and thereby promoting continuous visitation by multiple pollinator species. By consistently attracting diverse bee assemblages, these species help maintain connectivity among pollinators and plants within the network and may buffer interaction dynamics against temporal instability. Consequently, herbaceous plants exhibited significantly higher interactive roles than woody species, reinforcing the idea that the most influential species in mutualistic networks are those that provide abundant, generalist, and easily detectable resources that can be exploited by a broad spectrum of pollinators [10].

In addition to plant life form, visual floral traits also played a notable role in shaping plant importance within the interaction network. Among these traits, flower color (a key visual cue used by pollinators during foraging) showed a clear and complementary pattern to that observed for floral display and growth form. In our study, plant species with yellow flowers received proportionally more visits and exhibited higher interactive roles, a result consistent with experimental and observational evidence indicating that bees are particularly sensitive to wavelengths associated with yellow and ultraviolet-reflecting floral signals [65,66]. These colors are often highly conspicuous against vegetative backgrounds and can enhance long-distance detection as well as learning and memory during foraging bouts. Importantly, color preferences are not fixed and may vary depending on local ecological contexts, including plant dominance, community composition, and the availability of alternative floral resources [63]. In diverse plant communities, bees may preferentially visit colors that are either most rewarding or most abundant, whereas in species-poor systems, color preferences may be more flexible. Despite its clear influence on pollinator behavior, floral coloration remains relatively understudied in the context of interaction networks, in part because its evolutionary lability leads to high interspecific and intraspecific variation, making it difficult to draw broad generalizations across taxa and ecosystems [21]. By explicitly incorporating flower color into our analyses, our results highlight that visual floral traits, together with vegetative attributes such as life form, jointly contribute to defining the interactive role of plant species within bee–plant networks. These findings reinforce the idea that understanding why certain species emerge as highly connected and influential nodes requires a multidimensional trait-based perspective, rather than a focus on single traits in isolation. Such an integrative approach is essential for identifying plant species that disproportionately support pollinator diversity and network structure in heterogeneous tropical ecosystems.

In summary, our results demonstrate that plant functional traits differ markedly in their contribution to bee visitation patterns and to the structural roles that plant species occupy within interaction networks. Traits that signal resource abundance or accessibility (such as the number of surrounding flowers, flower size, and flower color) strongly increased visitation rates and promoted certain species to occupy higher interactive roles within the network. By enhancing detectability, accommodating bees of multiple body sizes, and providing consistent foraging opportunities across taxa, these traits increase both the frequency and diversity of plant–bee interactions. Importantly, the plant species that emerged as central nodes in our network tended to exhibit generalist and readily exploitable trait combinations, enabling interactions with a broad range of bee species [67]. By offering abundant, accessible, and predictable floral resources, these key species could be functioning as foundational components of the community, supporting high levels of bee diversity and reinforcing the overall connectivity and robustness of mutualistic networks. Such species may also play a critical buffering role by maintaining interaction pathways during periods of resource scarcity or environmental fluctuation, for instance. We acknowledge that our sampling represents a limited temporal window within the study site and that the identity of the most central plant species may change across seasons or years [68]. However, even if species turnover occurs, we expect that plants occupying central positions at other time frames will share similar functional traits with those identified here, particularly traits associated with high resource abundance and availability. Thus, while species identities may vary temporally, the trait combinations linked to high centrality are likely to remain consistent. Our findings emphasize the value of a multidimensional, trait-based framework for understanding why certain plant species disproportionately shape the structure and functioning of bee–plant interactions. Integrating both vegetative and floral traits provides a more mechanistic basis for predicting how plant–pollinator networks may respond to environmental change, including habitat alteration and climate variability. Finally, and from an applied perspective, this approach can help identify priority plant species for conservation, restoration, and management efforts aimed at sustaining pollinator diversity and preserving the stability of pollination services in tropical coastal ecosystems.

## Data Availability

No new data were created or analyzed in this study.
